# Social Media Engagement and Self-Esteem as Predictors of Adjustment Domains Among Adolescents With Body Dysmorphic Symptoms

**DOI:** 10.7759/cureus.95261

**Published:** 2025-10-23

**Authors:** Ravi P Pandey, Pramod Kumar, Vivek Singh, Tanya Sharma, Deepak Kumar, Shantesh Kumar Singh, Arun Kumar, Purnima Awasthi, FNU Nitu, Komal Bumra

**Affiliations:** 1 Psychology, Central University of Haryana, Mahendragarh, IND; 2 International Studies, Jawaharlal Nehru University, New Delhi, IND; 3 Psychology, Deen Dayal Upadhyaya (DDU) Gorakhpur University, Gorakhpur, IND; 4 Psychology, Banaras Hindu University, Varanasi, IND; 5 Psychology, Dr. Shree Krishna Sinha (SKS) Women's College, Motihari, IND

**Keywords:** adjustment, body dysmorphic disorder (bdd), body image, self esteem, social media engagement

## Abstract

Background: Social media has influenced the perception of beauty, attraction and body image among adolescents which is significantly regulated by socio-cultural factors. Individuals with low self-confidence and a strong desire for social acceptance tend to seek approval from their environment and become overly concerned with their physical appearance. Adolescents, who are more susceptible to insecurity and adjustment issues, are particularly vulnerable to the negative effects of social media.

Objective: This study comprises two objectives: 1) To investigate the relationship among self-esteem, social media engagement and adjustment in adolescents displaying symptoms of body dysmorphia. 2) To compare participants on the basis of gender and urban-rural divide on self-esteem, social media engagement and adjustment.

Participants: The study utilized a correlational design and t-test and collected self-reported data from 198 adolescents aged 13 to 19 years (M=17.43 years; SD=1.82).

Measures: The data were gathered using the Body Dysmorphic Disorder Questionnaire (BDDQ) screening questionnaire, Bell Adjustment Inventory, Social Media Engagement Scale (SMES) and State Self-Esteem Measure.

Result: Correlation analysis revealed a significant positive correlation between social networking usage and social adjustment and a negative association with other forms of adjustment, namely health, emotional and home. Regression analysis showed that state self-esteem negatively predicted home adjustment and health adjustment. Both state self-esteem and social self-esteem negatively predicted health adjustment. Conversely, social adjustment was positively predicted, while emotional adjustment was negatively predicted by social media engagement. The findings also revealed distinct patterns of adjustment and self-esteem based on gender and location differences in relation to social media engagement. The study concludes with a discussion of its theoretical and practical implications, along with methodological limitations.

## Introduction

Information and communication technologies (ICTs) have a large impact on everyone's life, and they influence social interaction and everyday communication [1,2]. This has an impact on one's psychological health in addition to how an individual communicates with others [3]. The use of social media websites is one of the most well-liked ways in which technology is used. The popularity of online social networks has driven the development of specialized platforms such as Facebook (Meta Platforms, Inc., Menlo Park, California, U.S.), Twitter (X Corp., Bastrop, Texas), Instagram (Meta Platforms, Inc., Menlo Park, California, U.S.) and Quora (Quora, Inc., Mountain View, California) [4]. With 330 million users, India is reported to have the most Facebook users in the world, followed by the United States with 210 million users [4]. These kinds of social media platforms are overloaded with filtered, edited and curated pictures, and the constant exposure to such non-realistic standards of body image can make adolescents to adopt those standards and become discontent with their own appearance.

Body dysmorphic disorder

Body dysmorphic disorder (BDD) is an obsessive and intrusive fixation on a minor or non-existent flaw in one’s appearance which is also associated with obsessive-compulsive disorder. It has a complex aetiology that includes neurochemical abnormalities, psychological impairment and cognitive deficiencies [5]. Studies have indicated that body dysmorphic disorder was found to be associated with greater delusional worries in children and adolescents than in adulthood [6]. These self-consciousness-related worries result in unfavourable feelings including guilt, worry and melancholy. These emotions are frequently so strong that affected persons avoid expressing them to healthcare experts for a fear of having their worries dismissed or trivialised [7]. Social media platforms, with their emphasis on curated images and ideals of beauty, promoting unrealistic beauty standards, manipulated and filtered images, and constant comparison, can exacerbate symptoms and trigger or worsen BDD in adolescent population.

Self-esteem and body dysmorphic disorder

Through the writings of authors like William James, Cooley and Mead, who started to place the notion of self at the centre of psychology [8-10], the idea of self-esteem came into common usage. Self-esteem was first conceptualized by William James in his seminal book, "The Principles of Psychology" [10]. James emphasised the connection between the happiness that comes from meeting one's fundamental requirements and how this leads to a sense of self-satisfaction, which increases one's appreciation of oneself. Self-esteem is the degree to which we evaluate our self positively, negatively, or even neutrally. Teenagers are at a rising stage of life when concern for one's self-evaluation and self-appraisal in relation to others is on the rise, compared to other age groups; hence, low self-esteem is especially associated with this age group. Individuals with BDD commonly experience disturbances in their self-esteem, as their persistent dissatisfaction and flaws in their body can lead to feelings of inadequacy, shame and low self-worth, exacerbating negative self-evaluations, contributing to a vicious cycle of self-criticism and diminished self-esteem [10].

Social networking and body dysmorphic disorder

The findings of the several studies reported that the use of social media sites increase the risk of body dysmorphic symptoms as well as maladjustment [11,12], whereas other researches revealed contradictory findings which suggested that the active use of social media enhanced self-esteem and well-being of the users. Issues related to body image during COVID-19 was also found linked to body dysmorphic disorder (BDD), such as a negative body image of oneself which exhibits through self-perception about the ugliness. A more concerning finding from a 2020 study revealed that using social media for longer periods of time, particularly on Snapchat and Instagram was linked to a higher incidence of BDD, with a notable increase in young participants [13].

Adjustment and body dysmorphic disorder

Despite the growing literature on gender similarities and differences in a variety of psychiatric disorders, this important aspect of body dysmorphic disorder (BDD) has received scant empirical attention, even though BDD is relatively common and severe [14,15]. To our knowledge, only two previous studies have examined this topic in BDD. One study, from the United States, contained 188 subjects (93 women and 95 men) from a BDD specialty program [14]; the other study, from Italy, contained 58 subjects (24 women and 34 men) who were consecutively enrolled as outpatients and had a chief complaint of BDD symptoms [14]. Both of the above-mentioned studies found far more gender similarities than differences in terms of most demographic characteristics, age at BDD onset, repetitive and safety behaviours, comorbidity, functional impairment and treatment received.

Self-esteem in the context of social networking sites

The relationship between self-esteem and social networking sites can be understood through various theoretical models. Mead's theory suggests that individuals with high self-esteem have a strong sense of "I," while those with lower self-esteem struggle with their self-concept, dominated by "Me." The concepts of upward and downward comparison also play a crucial role, with social media influencing self-esteem through these comparisons. Research by Valkenburg et al. (2017) [16] indicates that social media can positively impact self-esteem and life satisfaction. Additionally, studies highlight that self-perceived physical appearance significantly affects adolescents' self-esteem, particularly in girls, linking social media usage with body image concerns [16-20].

Self-esteem and adjustment

For young people and teenagers, self-esteem is a well-established predictor of positive adjustment. Another study shows that there are significant relationships between academic self-esteem and multiple indicators of academic accomplishment among high school students than between global self-esteem and academic achievement; media, peers and parents, as per sociological theory of body image, suggest the significance of appearance and the pressure to adhere to unrealistic body ideals which leads to appearance comparison [21-23].

In this study, researchers used four correlates of adjustment, home, health, social and emotional, in which home and social correlates related with environmental issues faced by individual and emotional correlates with cognitive aspect of individual. Present study is considered as having the predictive potential of global and particular self-esteem (i.e. performance, academic and social self-esteem) and related adjustment measures, such as overall, academic and social adjustment. With an increase in prevalence of social media in lives of everyone, adolescents are the most vulnerable populace for enduring the phase in which they are going through the consequences of social media usage, be it positive or negative.

Above-mentioned studies demonstrated a link between social media engagement and negative body image, depressive symptoms, anxiety and reduced social functioning, particularly when social media becomes a platform for cyberbullying or unhealthy comparison. However, it is important to acknowledge that research also suggests positive associations between social media engagement and positive body image and self-esteem in certain contexts.

Moreover, the current era is characterized by the dominance of social media, where the trend of sharing filtered pictures to appear attractive and garner likes, comments and compliments prevails. These activities significantly contribute to body image concerns among adolescents who scroll through social media platforms. This impact, in turn, influences their thoughts, daily activities, adjustment and performance in various areas such as academics, ultimately contributing to heightened stress levels.

The aim of the current study was to explore the association of social media engagement and self-esteem with the adjustment (home adjustment, health adjustment, social adjustment, emotional adjustment). The study aims to gain insights into the complex associations between social media engagement, self-esteem and adjustment among adolescents with symptoms of body dysmorphic symptoms.

The study also posited hypotheses, suggesting that the dimensions of self-esteem (social, performance and appearance self-esteem) would exhibit negative associations with adjustment (home, health, social and emotional adjustment). Additionally, the hypothesis suggested that social media engagement would be positively associated with poor home, health, social and emotional forms of adjustment among adolescents displaying symptoms of body dysmorphic disorder.

## Materials and methods

Participants

The data for this study were drawn from a total of 198 adolescents, who were initially selected through a convenient sampling method. Participants underwent screening using the Body Dysmorphic Disorder Questionnaire, resulting in the identification and exclusion of 270 individuals. Furthermore, 72 participants did not return the filled scales/questionnaires. Ultimately, 198 participants met the eligibility criteria and progressed to the next phase of the study. During the second step, these eligible participants completed the provided questionnaire, which has been included to measure social media engagement, self-esteem and the adjustments of the participants. Researchers collected the filled questionnaires from the participants and expressed gratitude for their participation. The data collection process encompassed obtaining assent, establishing rapport, providing clear instructions, conducting a screening process and collecting filled questionnaires from eligible participants.

Participants were sourced from various states in India, including Haryana, Delhi, Uttar Pradesh, Bihar and Rajasthan. Their ages ranged from 13 to 19 years, with a mean age of 17.43 years (SD=0.83). Convenient sampling method was applied to draw the data from the population. All 540 students were contacted in one time, out of which 270 did not exhibit the symptoms of body dysmorphic disorder and 72 did not fill the questionnaires properly. Participants were recruited by personally visiting the schools and contacting the school authorities and parents of adolescents. Parents of the participants were contacted via phone to obtain their consent, and written permission was obtained from the school authorities. Data were collected from the participants during lunchtime or when there were no scheduled classes. Authorities also supported in data collection by scheduling a special one-hour visit to the school.

Priori analysis

The bivariate normal model's a priori correlation was analysed using G*Power software version 3.1 (Heinrich-Heine-Universität Düsseldorf, Christian-Albrechts-Universität zu Kiel, and Universität Mannheim, Germany) to determine the sample size for the study. The medium effect size was calculated for both one and two tails. The output of the power analysis suggested that 115 participants would be required for one tail and 138 participants for two tails in the correlation analysis (Tables 1, 2).

**Table 1:** Priori analysis

Effect Size	Required Sample for One Tail	Required Sample for Two Tail
Medium (0.30)	115	138

**Table 2:** Demographic characteristics of participants included in the study

Demographic Variables	Category	N	Percent
Gender	Male	100	50.5
	Female	98	49.5
Residence	Urban	105	53
	Rural	93	47

Measures

Body Dysmorphic Disorder Questionnaire (BDDQ)

This questionnaire was developed by Phillips et al., (1995) [24]. It has four sections. First three section questions are dichotomous, and last and fourth section is based on three different time durations (a. less than one hour a day, b. one to three hours a day and c. more than three hours a day) thinking about appearance. Validity of this screening questionnaire was found a sensitivity of 94%, specificity of 90% and likelihood ratio of 9.4, that Body Dysmorphic Disorder Questionnaire (BDDQ) showed strong concurrent validity [23].

The participants of the study were adolescents who were screened using the Body Dysmorphic Disorder Questionnaire (BDDQ), which consists of four questions for screening purposes. The first question in the BDDQ has two sub-questions. The participants are required to answer "yes" or "no" to each sub-question. The first sub-question pertains to whether the participant worries about their physical appearance or looks. The second sub-question focuses on whether the participant has concerns about specific areas of their body or certain body parts that cause them distress. Participants are also asked to provide a written explanation of the actual causes of their worries about their physical appearance. The second question in the BDDQ is related to the main concerns the participant has regarding their physical appearance. The third question consists of four sub-questions that assess how the participant's physical appearance affects their daily life. The fourth and final question in the BDDQ evaluates the amount of time the participant spends each day thinking about their physical appearance.

Bell’s Adjustment Inventory (Student Form)

The Indian adaptation of Bell’s Adjustment Inventory (Student Form) has been used [25]. It contains 140 items related to the four adjustment domains of home, health, social and emotional. Each domain consists equal number of items, i.e. 35. A few examples of Home Adjustment items are: Have you ever had a firm idea of absconding the home? Do you ever feel that your parents are not satisfied with you? Examples of Health Adjustment items are: Do you often take medicines? Do you usually feel tired? Thus, in all analyses, higher scores reflect greater maladjustment. High scores on the scale indicate poor adjustment, whereas low scores indicate better adjustment in many focused areas and in overall adjustment. A score below 4 indicates a better adjustment level, while a score above 18 suggests a poorer adjustment level. The test was determined to be extremely reliable by computing split half and test-retest methods of reliability, which were found to be 0.84, 0.81, 0.87 and 0.89 by split-half method and 0.91, 0.90, 0.89 and 0.92 by test-retest method, respectively. The validity coefficient for the home, health, social and emotional areas of the inventory was 0.72, 0.79, 0.82 and 0.81, respectively [26].

State Self-Esteem Scale (SSES)

This scale is developed by Heatherton and Polivy (1991) [27]. It is a five-point (1=Not at All, A Little Bit, Somewhat, Very Much, 5=extremely) Likert scale. It contains 20 items with three dimensions such as performance self-esteem, social self-esteem and appearance self-esteem. Performance self-esteem component contains seven items, and the score of this component ranges from 7 to 35. Although this scale has good psychometric properties and has been used in various studies frequently, but recently, Chau (2023) [28] re-established the psychometric properties of the SSES. The SSES subscales’ Cronbach alphas ranged from 0.73 to 0.81.

Social Media Engagement Scale-Adolescents (SMES-A)

This scale was developed by Ni et al. (2020) [29]. It is a five-point (1=Strongly Disagree, and 5=Strongly Agree) Likert-type scale and contains 11 items. The score ranges from 11 to 77. High score of the scale indicates more engagement on social media, and low score indicates less engagement on social media. This scale is a composition of three factors such as affective engagement, behavioural engagement and cognitive engagement. Examples of the scale items are: Using social media is my daily habit; I browse social media whenever I have time. The Cronbach’s alpha coefficients of the three factors ranged from 0.709 to 0.804, and test-retest reliability was found to be 0.68.

Procedure

The data collection process for this study followed several steps. Firstly, formal permission was obtained from the head of the institution (government and private schools), and the informed consent was taken from parents of the participants by visiting their homes and contacting on phone call. An assent letter was used to get the approval of the students for participating in this study. Next, detailed instructions related to each questionnaire and inventory (social media engagement, self-esteem and adjustment inventory) were provided to the participants. The data collection process was completed in physical mode only to accommodate participants' preferences and convenience. The present study was a part of research dissertation in the Department of Psychology, Central University of Haryana. Departmental research committee of Department of Psychology has approved this research work in 2024. All aspects of the research were completed without harming participants or using any invasive techniques. Additionally, all ethical guidelines issued by the American Psychological Association (APA) have been followed diligently throughout the entire process, from data collection to writing the manuscript.

## Results

Statistical analysis

This section presents the study's findings, which involved computing descriptive statistics (mean and standard deviation) and applying correlation on scores obtained from the social media engagement, self-esteem and Bell Adjustment Inventory. The variables analysed encompass state self-esteem, performance self-esteem and appearance self-esteem (dimensions of self-esteem), as well as home, health, emotional and social health (forms of adjustments), and the screening tool of the Body Dysmorphic Disorder Questionnaire.

The results of the correlation analysis for the study variables are outlined in Table 2. Tables 3-7 present additional analysis, incorporating the outcomes of multiple hierarchical regression analysis using predictor and criterion variables. The hierarchy is structured based on the strength of correlation coefficients, with predictors entered block-wise into the regression model using the enter method.

**Table 3:** Correlation coefficients of social media engagement, state self-esteem (performance self-esteem, social self-esteem and appearance self-esteem) with the forms of adjustments (home, health, emotional and social) *p<0.05, **p<0.01. HoA: home adjustment, HeA: health adjustment, SA: social adjustment, EA: emotional adjustment, SME: social media engagement, PSE: performance self-esteem, SoSE: social self-esteem, ASE: appearance self-esteem.

Variables	HoA	HeA	SA	EA	SME
SME	0.10	-0.01	0.20**	0.03	-
PSE	-0.13	-0.14*	-0.13	-0.16*	-0.33**
SoSE	-0.14*	-0.22**	-0.15*	-0.03	-0.32**
ASE	-0.004	-0.07	-0.08	-0.18*	-0.15*

**Table 4:** Multiple hierarchical regression analysis on state self-esteem (performance, social and appearance self-esteem) and social media engagement as predictors and home adjustment (dimension of adjustments) as criterion variable *p<0.05. SME: social media engagement, PSE: performance self-esteem, SoSE: social self-esteem, ASE: appearance self-esteem, SE: standard error, B: unstandardized beta, β: standardized beta, LL: lower limit, UL: upper limit, VIF: variance inflation factor.

Variables	B	95% CI for B LL UL		SE B	β	R^2^	ΔR^2^	Collinearity Diagnostics (VIF)	p-value
Step-1									
SoSE	-0.20*	0.40	0.05	0.10	-0.14*	0.020	0.020*	1.00	0.044
Step-2									
SoSE	-0.15	-0.37	0.07	0.11	-0.10	0.026	0.005	1.26	0.296
PSE	-0.16	-0.45	0.14	0.15	-0.08			1.26	
Step-3									
SoSE	-0.013	-0.36	0.09	0.11	-0.09	0.028	0.002	1.31	0.570
PSE	-0.13	-0.44	0.17	0.15	-0.07			1.32	
SME	0.02	-0.06	0.11	0.04	0.04			1.16	
Step-4									
SoSE	-0.13	-0.35	0.09	0.11	-0.09	0.052	0.024*	1.31	0.028
PSE	-0.55	-1.1	-0.08	0.24	-0.29			3.30	
SME	0.07	-0.08	0.09	0.04	0.01			1.21	
ASE	0.53*	0.06	0.99	0.24	0.26*			2.81	

**Table 5:** Multiple hierarchical regression analysis on state self-esteem (performance, social and appearance self-esteem) and social media engagement as predictors and health adjustment (dimension of adjustments) as criterion variable *p<0.05, **p<0.01. SME: social media engagement, PSE: performance self-esteem, SoSE: social self-esteem, ASE: appearance self-esteem, SE: standard error, B: unstandardized beta, β: standardized beta, LL: lower limit, UL: upper limit, VIF: variance inflation factor.

Variables	B	95% CI for B LL UL		SE B	β	R^2^	ΔR^2^	Collinearity Diagnostics (VIF)	p-value
Step-1									
SoSE	-0.32**	-0.52	-0.11	0.10	-0.22**	0.046	0.046	1.00	0.002
Step-2									
SoSE	-0.28*	-0.51	-0.05	0.12	-0.19*	0.049	0.02	1.26	0.477
PSE	-0.11	-0.42	0.19	0.16	-0.06			1.26	
Step-3									
SoSE	-0.27*	-0.50	-0.05	0.12	-0.19*	0.053	0.04	1.26	0.366
PSE	-0.28	-0.76	0.20	0.24	-0.14			3.04	
ASE	0.22	-0.26	0.70	0.26	0.10			2.71	
Step-4									
SoSE	-0.31**	-0.54	-0.08	0.12	-0.21	0.064	0.011	1.31	0.136
PSE	-0.38	-0.88	0.11	0.25	-0.19			3.30	
ASE	0.29	-0.20	0.78	0.25	0.14			2.81	
SME	-0.067	-0.16	0.21	0.05	-0.12*			1.21	

**Table 6:** Multiple hierarchical regression analysis on state self-esteem (performance, social and appearance self-esteem) and social media engagement as predictors and social adjustment (dimension of adjustments) as criterion variable *p<0.05, **p<0.01. SME: social media engagement, PSE: performance self-esteem, SoSE: social self-esteem, ASE: appearance self-esteem, SE: standard error, B: unstandardized beta, β: standardized beta, LL: lower limit, UL: upper limit, VIF: variance inflation factor.

Variables	B	95% CI for B LL UL		SE, B	β	R^2^	ΔR^2^	Collinearity Diagnostics (VIF)	p-value
Step-1									
SME	0.08	0.03	0.15	0.03	-0.20**	0.041	0.041	1.00	0.004
Step-2									
SME	0.08	0.01	0.14	0.03	-0.17*	0.050	0.08	1.11	0.189
SoSE	-0.11	-0.26	0.05	0.08	-0.10			1.11	
Step-3									
SME	-0.07*	0.007	0.14	0.03	-0.16	0.051	0.01	1.16	0.602
SoSE	-0.09	-0.26	0.08	0.09	-0.08			1.31	
PSE	-0.06	-0.29	0.17	0.12	-0.04			1.32	
Step-4									
SME	0.07	0.005	0.14	0.03	0.16	0.051	0.00	1.21	0.935
SoSE	-0.09	-0.26	0.06	0.09	-0.08			1.31	
PSE	-0.07	-0.42	0.30	0.19	-0.05			3.30	
ASE	0.02	-0.35	0.38	0.18	0.01			2.81	

**Table 7:** Multiple hierarchical regression analysis on state self-esteem (performance, social and appearance self-esteem) and social media engagement as predictors and emotional adjustment (dimension of adjustments) as criterion variable *p<0.05. SME: social media engagement, PSE: performance self-esteem, SoSE: social self-esteem, ASE: appearance self-esteem, SE: standard error, B: unstandardized beta, β: standardized beta, LL: lower limit, UL: upper limit, VIF: variance inflation factor.

Variables	B	95% CI for B LL UL		SE B	β	R^2^	ΔR^2^	Collinearity Diagnostics (VIF)	p-value
Step-1									
ASE	-0.40*	-0.71	-0.09	0.16	-0.18*	0.033	0.033	1.00	0.011
Step-2									
ASE	-0.34	-0.85	0.17	0.26	-0.15*	0.033	0.01	2.71	0.750
PSE	-0.08	-0.55	0.40	0.24	-0.04			2.71	
Step-3									
ASE	-0.33	-0.85	0.19	0.26	-0.15	0.035	0.00	2.81	0.931
PSE	-0.09	-0.60	0.42	0.26	-0.04			3.30	
SME	-0.004	-0.10	0.090	0.05	-0.07			1.17	
Step-4									
ASE	-0.33	-0.85	0.19	0.26	0.15	0.035	0.02	2.81	0.570
PSE	-0.12	-0.65	0.40	0.27	-0.06			3.30	
SME	0.01	-0.09	0.09	0.05	0.02			1.20	
SoSE	0.07	-0.18	0.32	0.13	0.05			1.31	

Table 3 highlights that social media engagement was positively correlated with social adjustment scores (r=0.20, p<0.01), indicating that higher engagement was associated with poorer social adjustment. Social media engagement also demonstrates significant negative associations with performance self-esteem (r=-0.33, p<0.01), social self-esteem (r=-0.32, p<0.01) and appearance self-esteem (r=-0.15, p<0.05), as measured by the State Self-Esteem Scale. Moreover, Table 3 reveals that social self-esteem exhibits significant negative correlations with high scores on three forms of adjustment: home (r=-0.14, p<0.05), health (r=-0.22, p<0.01) and social adjustment (r=-0.15, p<0.05), the latter being the final factor of state self-esteem. Additionally, appearance self-esteem demonstrates a significant negative correlation solely with poor emotional adjustment (r=-0.18, p<0.05).

Tables 4-7 present the results of multiple hierarchical regression analyses examining the impact of predictor variables, specifically the dimensions of state self-esteem (social, performance and appearance self-esteem), on criterion variables representing domains of adjustments (home, health, social and emotional adjustment). The application of multiple hierarchical analysis aligns with previous research on these variables, and predictor variables were sequentially entered in block-wise fashion into the regression model. The enter method was employed to complete the analysis, with predictor variables prioritized based on their importance and correlation coefficients determined through Pearson product-moment correlation.

Table 4 indicates that social self-esteem significantly contributed to 2.00% variance in adolescent home adjustments. Notably, social self-esteem negatively predicted poor home adjustments (β=-0.14, p<0.05). While performance self-esteem did not make a significant individual contribution to home adjustments (approximately 0.05% of variance), the combination of social self-esteem and performance self-esteem jointly made a significant contribution, leading to a reduction in poor home adjustments among adolescents with symptoms of body dysmorphic disorder (β=-0.08, -0.10).

Moving to the next block, social media engagement was introduced, but it did not significantly contribute to home adjustments (approximately 0.02% variance). The beta values were not statistically significant, although they indicated a negative direction, suggesting a potential reduction in poor home adjustments among adolescents with symptoms of body dysmorphic disorder. In the fourth step, appearance self-esteem was introduced into the regression model, revealing a significant contribution of 2.4% of variance in adolescent home adjustments with body dysmorphic disorder. Importantly, appearance self-esteem positively impacted and reduced home adjustments among adolescents (β=0.26, p<0.05), with body dysmorphic disorder.

Table 5 reveals that social self-esteem significantly contributed to 4.6% of variance in the scores of poor health adjustments among adolescents, and it negatively predicted poor health adjustments with body dysmorphic disorder (β=-0.22, p<0.01). While performance self-esteem did not individually contribute significantly to poor health adjustments (approximately 0.02% of variance), the combined effect of social self-esteem and performance self-esteem made a significant contribution, leading to a reduction in poor health adjustments among adolescents (β=-0.19, p<0.01) with body dysmorphic disorder.

Entering appearance self-esteem in the subsequent block did not yield a significant contribution, accounting for approximately 0.04% of variance in poor health adjustments. However, the beta value was significant and indicated a negative direction for poor health adjustments among adolescents (β=-0.19, p<0.01), with body dysmorphic disorder. Subsequently, social media engagement was introduced into the regression model, revealing that this predictor did not make a significant contribution to poor health adjustments (explaining only 1.1% of variance). Importantly, when considering all three dimensions of self-esteem and social media engagement together, they made a significant combined contribution, explaining 6.4% of the variance in poor health adjustments among adolescents with symptoms of body dysmorphic disorder. Social media engagement also played a role in reducing poor health adjustments among adolescents (β=-0.12, p<0.05).

Table 6 illustrates that social media engagement significantly contributed to 4.1% of the variance in the scores of social adjustments among adolescents, and this predictor negatively predicted social adjustments (β=-0.20, p<0.01) with body dysmorphic disorder. Social self-esteem, in isolation, did not make a significant contribution to social adjustments, accounting for approximately 0.08% of variance. However, when combined with social media engagement, social self-esteem made a significant contribution and positively impacted the social adjustments of adolescents (β=0.17, p<0.01) with body dysmorphic disorder.

Entering performance self-esteem in the subsequent block did not yield a significant contribution, accounting for approximately 0.01% of variance in the measure of social adjustments. The beta value was not significant and indicated a negative direction for social adjustments among adolescents with symptoms of body dysmorphic disorder. Nevertheless, when considering social media engagement, social self-esteem and performance self-esteem together, they made a significant combined contribution to social adjustments. Appearance self-esteem did not contribute to social adjustments, explaining 0.00% of the variance. However, when all three dimensions of self-esteem and social media engagement were considered together, they made a significant contribution, explaining 5.1% of the variance in social adjustments among adolescents with symptoms of body dysmorphic disorder. It can be inferred that appearance self-esteem did not independently contribute to the poor social adjustments of adolescents.

Table 7 indicates that appearance self-esteem significantly contributed to 3.3% of the variance in the scores of emotional adjustments among adolescents, and this predictor negatively predicted poor emotional adjustments (β=-0.18, p<0.05). Performance self-esteem, when considered independently, did not make a significant contribution to emotional adjustments, accounting for approximately 0.01% of variance. However, when appearance and performance self-esteem were combined, they made a significant joint contribution (3.3%), leading to a reduction in poor emotional adjustments among adolescents, as indicated by the negative beta value.

In the subsequent block, social media engagement was introduced, but it did not make a significant contribution, explaining approximately 0.00% of variance in emotional adjustment. The beta value was not significant and indicated a negative direction for emotional adjustments among adolescents with symptoms of body dysmorphic disorder. Nonetheless, when considering social media engagement, appearance self-esteem and performance self-esteem together, they made a significant combined contribution to emotional adjustments. Social self-esteem did not make a significant individual contribution to emotional adjustments, accounting for only 0.2% of variance in emotional adjustment scores. Nevertheless, when all three dimensions of self-esteem and social media engagement were considered together, they made a significant combined contribution, explaining 3.5% of the variance in emotional adjustments among adolescents with symptoms of body dysmorphic disorder.

## Discussion

The present study was conducted with an aim to explore the role of self-esteem and social media engagement in the different domains of adjustment involving home adjustment, health adjustment, social adjustment and emotional adjustment. The finding of the study was not found consistent with previous research, indicating that higher levels of social media usage were associated with higher levels of social capital, including improved social adjustment which might be due to a greater social support, better social skills and a sense of belongingness one experiences while interacting with others on social media [30,31]. In this study, the intensity of social media engagement appears to be significantly and negatively related to performance, social, appearance and overall state self-esteem which is supported by social comparison theory by Festinger (1954) [32], according to which individuals have a predisposition for comparing themselves with other people for self-assessment, and this behaviour especially peaks throughout adolescence. Adolescents easily participate in social comparisons by using social media, and failing to meet a significant appearance criterion predicts lower self-esteem and high body dissatisfaction, which is very commonly seen in adolescents having a preoccupation with perceived flaws or defects in one's physical appearance, as in body dysmorphia symptoms. Our results provide consistent evidence to support prior research [33] that such negative associations exist, with a slightly greater magnitude in performance and social self-esteem factors of state self-esteem.

An interesting finding of the study is the significant negative correlation of factors of state self-esteem with all forms of adjustment that is home, health, social and emotional adjustment, with the magnitude of correlation being the highest in home, health and emotional adjustment, and the lowest in social adjustment. These findings support the popular view that increased global, academic and social self-esteem predict greater academic, social adjustment and overall wellness [34,35]. Another explanation of this might be that an existing negative adjustment pattern which appears in BDD symptomatic sample could be positively related with the stable self-esteem which is the trait aspect of self-esteem rather than the transient which is the state aspect. Effect of social media use in altering these factors, since prior studies have shown passive use of social media’s negative association with self-esteem [36], which in turn has been found to be negatively correlated with domains of adjustment in the present study.

The findings of the regression analysis are also in agreement with previous literature of relation between self-esteem and adjustment correlates conducted on a sample of non-clinical population. Explanation for this can be the role of social media use in affecting these variables simultaneously, wherein studies have supported that passive use (only liking and commenting on other’s posts) of social media is negatively related with self-esteem. Undoubtedly, some other dynamics might be at play for this population which affect relationship between self-esteem and adjustment correlates that need to be further explored.

Contrary to some expectations, social media engagement was associated with poorer social adjustment in this symptomatic sample. It is worth noting that a growing body of research has considered “active” versus “passive” kinds of social media use on well-being, but it has become crucial to expand the domains of social media use into “self-oriented” use in which the individuals post photos or status updates in their account, and “other oriented” use in which the individuals are engaged in liking posts of others, especially while examining the effects on appearance self-esteem, because posting one’s own pictures as an “active user” versus just hitting likes or posting comments on others’ posts like a “passive user” appears to impact judgements about one’s own appearance differently. The findings extend prior research [34,37], by suggesting that social media engagement positively predicted social adjustment in body dysmorphic symptomatic adolescents.

Sociocultural models of the development of body dissatisfaction and eating disorder such as the tripartite model and the dual pathway model provide a framework to guide and understand the factors relevant to development of dissatisfaction. According to this model, appearance pressure that comes from peers, family and media and psychological processes contribute to the emergence and maintenance of body dissatisfaction. In relation to media, social media use is becoming highly relevant for young adolescents compared with traditional media, with Instagram and Snapchat used more frequently than other platforms. Over the past decade, the total number of hours per day that early adolescents devoted to social media had risen and the percentage of those who used social media on a daily basis had almost doubled. Therefore, investigating the influence of social media, rather than traditional media, on body dissatisfaction may be more reflective of the media environment in which adolescents are engaged.

Limitations

The present study has several limitations that need to be acknowledged. Firstly, it was a cross-sectional study, and as such, the data collected does not provide insights into causal relationships between social networking usage, self-esteem and forms of adjustment. Secondly, the research did not assess different types of social media use, such as active and passive engagement or truly passive activities like browsing. Additionally, future work should consider incorporating potential moderators, such as types of posts and feedback received, as well as mediators like appearance comparison, which could be examined in subsequent studies.

Thirdly, the study's findings should be interpreted cautiously, and there is a need for confirmation as they did not align consistently with prior research outcomes conducted on a general population. Challenges were encountered in the study population due to the BDD screening procedure employed, and the response rate was lower among the adolescent age group during data collection. Measures used in this study has not been standardized on study population of this study. This may impact the generalizability of the findings.

Implications

The study has undertaken an effort to elucidate both the positive and negative impacts of social media engagement on the self-esteem of adolescents exhibiting symptoms of body dysmorphic disorder and different domains of adjustments. Understanding these intricate processes holds crucial significance for the formulation and implementation of prevention and intervention strategies within families, society and the community to overcome the issues related to body image and body dysmorphic disorder. Formal media literacy programs may be initiated to regulate and channelize social networking usage in a beneficial manner among adolescents and so that problems like adjustments can be improved and disorders like BDD can be prevented.

Furthermore, the study has the potential to contribute to the dissemination of awareness regarding body dysmorphic disorder, along with its associated risks, preventive measures, and protective factors. To extend the impact on a broader scale, skill development programs focusing on areas such as interpersonal skills, confidence training, tolerance building, effective use of social media and awareness programs emphasizing good eating and sleeping habits, the benefits of a nutritious diet, the advantages of regular physical exercise and the maintenance of a positive body image can be planned for the adolescents and children.

## Conclusions

The present study provides valuable insights into the complex interplay between self-esteem, social media engagement and various domains of adjustment home, health, social and emotional among adolescents exhibiting symptoms of body dysmorphic disorder (BDD). The findings reveal that higher levels of social media engagement are significantly and negatively associated with performance, social, appearance and overall state self-esteem. This pattern supports social comparison theory, emphasizing the vulnerability of adolescents to appearance-based comparisons in online environments. The negative correlation between factors of state self-esteem and adjustment dimensions further highlights that lower self-esteem is linked with poorer adjustment, particularly in home, health and emotional domains.

Contrary to earlier research suggesting potential social benefits of online connectivity, this study indicates that excessive or passive social media use may impair self-perception and hinder adaptive functioning. These outcomes underscore the necessity of distinguishing between different modes of social media use active, passive, self-oriented and other-oriented, to better understand their psychological consequences. The results also suggest that sociocultural pressures, reinforced through digital interactions, contribute to heightened appearance concerns and diminished self-worth among adolescents.

Overall, the study emphasizes the urgent need for preventive and corrective measures, including media literacy education, structured awareness programs and skill development initiatives aimed at promoting healthy social media practices and fostering positive body image. By addressing maladaptive comparison tendencies and encouraging balanced digital engagement, such interventions can support adolescents in achieving better self-esteem, emotional stability, and holistic adjustment.
